# Comparison of Antibody Isotype Response to *Angiostrongylus cantonensis* in Experimentally Infected Rats (*Rattus norvegicus*) Using Hawai’i 31 kDa Antigen in an Indirect ELISA

**DOI:** 10.3390/pathogens12040625

**Published:** 2023-04-21

**Authors:** Argon Steel, Lisa Kaluna, John Jacob, Susan Jarvi

**Affiliations:** Department of Pharmaceutical Sciences, Daniel K. Inouye College of Pharmacy, University of Hawai’i at Hilo, 200 West Kawili St., Hilo, HI 96720, USA

**Keywords:** *Angiostrongylus cantonensis*, angiostrongyliasis, rat lungworm, serological detection, ELISA, 31 kDa

## Abstract

Neuroangiostrongyliasis (NAS) is an emerging tropical disease in humans and some animals which is caused by infection with the parasitic nematode *Angiostrongylus cantonensis*. It is the leading cause of eosinophilic meningitis worldwide. Diagnoses in humans and susceptible animals are generally presumptive and easily confused with other central nervous system disorders. The 31 kDa antigen is currently the only NAS immunodiagnostic assay that has achieved 100% sensitivity. However, little is known about the humoral immune response against the 31 kDa antigen in NAS infections, which would be critical for widespread adoption of this assay. We used the Hawai’i 31 kDa isolate in an indirect ELISA assay to confirm the presence of immunoglobulin IgG, IgM, IgA, and IgE isotypes in six-week post-infection plasma from lab-reared rats infected with 50 live, third-stage, *A. cantonensis* larvae isolated from a wild *Parmarion martensi* semi-slug. Our results confirmed the presence of all four isotypes against the Hawaii 31 kDa isolate, with sensitivity ranging from 22–100%. The IgG isotype showed 100% sensitivity in detecting *A. cantonensis* infection, which validates the use of IgG indirect ELISA with 31 kDa antigen as an effective immunodiagnostic assay for rats six weeks post-infection. Given each isotype may be present at different times during NAS infections, our data provides preliminary information on the humoral immune response to *A. cantonensis* infection in lab-reared rats and serves as a baseline for future studies.

## 1. Introduction

The nematode *Angiostrongylus cantonensis* (rat lungworm) is a food and water-borne zoonotic parasite causing neuroangiostrongyliasis (NAS), an emerging tropical infectious disease and the leading cause of eosinophilic meningitis worldwide [1,2]. In the life cycle of *A. cantonensis*, rats are the obligate definitive host, with adult worms reproducing in the pulmonary arteries, and gastropods are the obligate intermediate host [3]. Humans and other mammals, such as horses and dogs, can be accidental hosts, in which *A. cantonensis* does not reproduce but does invade the central nervous system (CNS), causing painful and devastating symptoms [3,4,5,6]. Symptoms of NAS are frequently mistaken for other central nervous system disorders, leading to frequent misdiagnoses in humans and animals [7,8].

A reliable laboratory diagnostic assay that can be used in conjunction with patient history and clinical symptoms has been a longstanding goal for NAS. The gold standard for diagnosis of *A. cantonensis* infection is the microscopic identification of whole larvae in the cerebrospinal fluid (CSF) [9]. With advances in technology, the Centers for Disease Control and Prevention (CDC) currently tests human CSF for *A. cantonensis* DNA using a real-time polymerase chain reaction (PCR) assay [10], and a few laboratories offer real-time PCR testing for animals. The most recent real-time PCR assay for NAS [11] meets important laboratory diagnostic criteria, such as having high sensitivity, and will likely be the assay of choice in the near future.

Nevertheless, while no laboratory diagnostic is perfect in all circumstances, numerous problems plague both the identification of whole larvae in CSF and real-time PCR, which complicate testing and delay or prevent patient diagnosis. For example, collection of CSF can be difficult to obtain, as some patients, doctors, or veterinarians are hesitant to perform a lumbar puncture. Both microscopic identification and real-time PCR have a high failure rate due to the scarcity of their targets and often require repeated lumbar punctures for positive identification [9]. As a result, most diagnoses are presumptively based on compatible history, characteristic physical findings, and evidence of high eosinophil counts [7,12]; thus, ongoing research is searching for a less invasive and more robust laboratory diagnostic. As an immunodiagnostic test, the measurement of antibody titers has the potential for high sensitivity while also overcoming some barriers given that blood collection is less invasive, antibodies can reach levels easily detected, and the advantage that previous exposure to the pathogen can be retrospectively detected.

For decades, a variety of antibody detection methods have been investigated for NAS, with most research focused on the enzyme-linked immunosorbent assay (ELISA). For example, Cross [13] developed an ELISA assay using *A. cantonensis* crude antigen isolated from fourth-stage larvae recovered from rat brains. When tested against other helminths, the ELISA values for *A. cantonensis* were higher, yet cross-reactivity was still significant. Subsequent research has been directed toward finding discrete antigens with greater specificity through various methods of purification of *A. cantonensis* proteins [14,15,16]. A number of these studies found that serum antibodies from human patients with NAS specifically recognized 29 kDa and 31 kDa proteins present in crude antigen of adult worms, with further research showing greater specificity for the 31 kDa protein [6,12]. After purifying the 31 kDa antigen using electrophoresis in a 12% SDS-polyacrylamide gel, Eamsobhana et al. [17] were able to achieve 100% sensitivity and specificity when testing serum from human patients with active *A. cantonensis* infections. Some research suggests that native antigens isolated from geographically local *A. cantonensis* may enhance the detection of exposure to the parasite or prior NAS infections, particularly in non-endemic areas [10,18]. Despite having been validated over a decade ago, there is a lack of basic immunological information about the humoral immune response to the 31 kDa antigen that is pertinent for a robust laboratory diagnostic. Knowing how antibody isotype titers change over time, in different tissues, following various infection loads, or in different hosts would likely be helpful to distinguish either early diagnosis or between active and prior infections.

Investigations into the NAS humoral immune response are most easily conducted using a laboratory-reared animal model to eliminate or reduce cross-reactivity from prior infections, as well as to gain the ability to control the dose and timing of infection. While the definitive host (the rat) may have different humoral immune responses to NAS than accidental hosts (humans or other mammals), the rat is a valuable and widely used model for investigating humoral immune response and assay validation. Likewise, while the IgG antibody is the most widely used isotype for ELISA assays in both NAS and non-NAS infections, other isotypes may be informative as a laboratory diagnostic, particularly early in a NAS infection when IgG levels are lower [19,20,21,22]. IgM is the first to appear following initial exposure to an antigen and might be particularly informative for early NAS diagnosis. IgA plays a role in mucous membranes and could be activated early in an infection when *A. cantonensis* is in the gastrointestinal tract. As a critical part of the immune defense against parasitic worms, IgE may also be informative. In fact, immunoglobins G, M, A, and E are all detectable in the sera and CSF of humans with acute, natural NAS infections [16,23]. In this study, we optimized methods developed for human sera [24] to confirm the presence of IgG, IgM, IgE, and IgA isotypes in six-week post infection (PI) rat plasma against the Hawaii 31 kDa isolate using an indirect ELISA assay.

## 2. Materials and Methods

### 2.1. Rat Plasma

The plasma for this study was collected from rats used to validate the use of propidium iodide stain as an in vitro death assay [25]. There were several advantages of using this plasma source. First, laboratory strains of rats were used in the study instead of wild rats, minimizing the likelihood of cross-reactivity or acquired immunity resulting from prior infections. Additionally, appropriate experimental groups were selected for the ELISA study, enabling the inclusion of both positive and negative controls, a suitable number of replicates within experimental groups, and sufficient plasma for testing. While plasma collection was limited to the six-week PI necropsy, this time point is a good starting point for ELISA validation and testing in rats, as IgG titers are expected to be high. This is because *A. cantonensis* completes its life cycle by this time, and IgG against crude antigen is typically present between 10–30 days and peaks by 50–60 days PI [19,20,21,22].

Briefly, 40 7-month-old Wistar IGC outbred laboratory strains of *Rattus norvegicus* were obtained from Charles River Labs (Raleigh, NC, USA) and maintained as described at the USDA-APHIS Wildlife Services National Wildlife Research Center (NWRC) Hawai’i Field Station, Hilo, HI, USA. Rats were gavaged under sedation as follows: Uninfected control rats were fed 1 mL of dH_2_O only, without larvae, while other rats were gavaged with 1 mL of dH_2_O containing 50 *A. cantonensis* L3, either live or dead, stained with propidium iodide or unstained. Thus, experimental groups consisted of: (1) live-stained larvae (3 male/2 female rats); (2) live-unstained larvae (2 male/2 female rats; one female was found dead in the cage, 40 days PI and before euthanasia and cardiac bleed); (3) killed-stained larvae (5 male/5 female rats); (4) killed-unstained larvae (5 male/5 female rats); and (5) uninfected controls (5 male/5 female rats) for a total of 39 rats. All *A. cantonensis* larvae were obtained from a single semi-slug (*Parmarion martensi*) collected from the University of Hawai’i at Hilo campus.

Rats were humanely euthanized six weeks PI, and necropsies were conducted as described [25]. Briefly, following euthanasia, whole blood samples (up to 2 mL) were collected via cardiac puncture of the right ventricle using a tuberculin syringe and transferred to heparinized collection vials for plasma isolation, transferred to sterile screw-capped tubes, and stored at −80 °C until testing with ELISA. In addition, selected organs were dissected: heart and lung were examined for adult *A. cantonensis*, the brain for the presence of larvae, and the lungs were examined for evidence of granulation (see Supplementary Materials Data Table S1).

### 2.2. Hawai’i 31 kDa Isolation

The Hawai’i 31 kDa isolate prepared by Jarvi et al. [26] with methods based on Eamsobhana et al. [17,27] was used for ELISA testing in this study. Briefly, adult female worms were harvested from the heart and lung tissue of infected rats collected from East Hawai’i Island between January through February 2017, washed with 1X PBS buffer, and stored at −80 °C in 1X protease inhibitor (Biochem Cocktail set V EDTA-Free, Thermo Scientific, Waltham, MA, USA) in 0.01 M PBS (Life Technologies, Grand Island, NY, USA). Frozen worms were homogenized manually in 1X protease inhibitor diluted in 1X PBS with a glass homogenizer, then sonicated (QSonica) on ice between 3 to 10 times in 3 s intervals with a 20 s rest period between each cycle to prevent overheating. The homogenized sample was stored at 4 °C overnight, centrifuged, and collected as soluble antigen (supernatant). The recovered antigen was quantified using a Coomassie Plus (Bradford) Assay Kit (ThermoFisher Scientific, Waltham, MA USA), then separated on a series of 12% SDS-polyacrylamide gels using electroelution to isolate the 31 kDa targeted antigen. Sections of gel containing the 31 kDa proteins were identified using a Hi-Mark^TM^ Pre-stained Protein Standard ladder, and these gel sections were excised manually. The gel slice was minced and then eluted with a Model 422 Electro-Eluter (Bio-Rad Laboratories, Hercules, CA, USA). Electroeluated protein was desalted and concentrated by ultrafiltration using Amicon Ultra-2 Centrifugal Filter Devices (MilliPore Sigma, Burlington, MA, USA) and quantitated using High Sensitivity Protein 250 chips in an Agilent 2100 Bioanalyzer System (Agilent Technologies, Santa Clara, CA, USA) as having a protein concentration of 1.69 µg/µL. Quantified proteins were pooled and stored in 1.5 mL low protein binding microcentrifuge tubes (Eppendorf, Hauppauge, NY, USA) at −80 °C.

### 2.3. Indirect ELISA

ELISA was based on the methods described in Jarvi et al. [26], with some modifications. Antigen, plasma, and conjugate concentrations were determined using checkerboard titration. Plasma from rats fed live-unstained larvae was used as positive controls, and plasma from uninfected rats was used as negative controls; infection status was confirmed for all experimental groups through necropsy. Primary antibodies were derived via titration of positive and negative plasma controls with a range of 1:50 to 1:1600, resulting in final concentrations of 1:100 for IgG and IgM and 1:50 for IgA and IgE. Concentrations of secondary antibody (all conjugated with horseradish peroxidase) were derived from titration from an original range of 1:450 to 1:100,000, with final concentrations of 1:5000 for IgG-Fc Fragment (Bethyl Labs, A110-136P, Lot 30) and IgM (Bethyl Labs, A110-100P, Lot: 46) and 1:1250 for IgA (Bethyl Labs, A110-102P, Lot: 40) and IgE (Invitrogen, No. SA5-10256, Lot XE3581801). Antigen concentrations of 0.25 µg/well for IgG and IgM and 0.5 µg/well for IgA and IgE and were derived from a titration of 1.0 to 0.0313 µg/well, quantitated from 31 kDa antigen with a concentration of 1.69 µg/µL (see above).

Each plate included at least two positive controls, two negative controls, and a single carbonate buffer control or blank (without antigen). All samples and controls were run in triplicate. Flat bottom 96-well Immulon 4HBX microtiter plates (ThermoScientific) were initially coated with 31 kDa antigen derived from *A. cantonensis* and diluted with 0.05 M BupH carbonate–bicarbonate buffer (ThermoScientific, #28382) at pH 9.4 and refrigerated overnight at 4 °C. All washing steps were conducted on a 405 Select TS microplate washer (BioTek, Winooski, VT, USA). After coating, plates were washed four times with 300 μL PBS–0.05% Tween 20 (PBS-T) (pH 7.4) with a 2 min pause after every other wash. Plates were then blocked (125 μL/well) with 5% BLOTTO (nonfat dry milk powder) in PBS-Tween (PBS-T) for 2 h at room temperature with gentle rocking. The blocking solution was removed with no washing, then plasma samples diluted with 2.5% BLOTTO in PBS-T were added to appropriate wells (100 μL/well) and incubated for 2 h at 37 °C with gentle shaking. After incubation with primary antibody, plates were washed six times with 300 μL PBS-T, with a 2 min pause after every other wash. Horseradish peroxidase (HRP)-conjugated goat anti-rat secondary antibody diluted in 2.5% BLOTTO in PBS-T was added (100 μL/well), and plates were incubated for 1 h at 37 °C with gentle shaking. Plates were then washed six times with PBS-T, pausing every other wash. TMB-solubilized substrate solution (TMB One^®^; Promega, Madison, WI, USA) for HRP was added (100 µL/well). Absorbance was monitored on an µQuant microplate reader (BioTek) at 650 nm and is reported as optical density (OD). Upon the positive controls reaching a maximum OD of ≤0.6, the reaction was stopped with 1 N HCl (100 µL/well), allowed to equilibrate for 5 min with gentle rocking, then OD read at 450 nm.

### 2.4. Indirect ELISA Data Analysis

The cutoff value to distinguish positive vs. negative infection status was determined for each immunoglobulin class by taking the mean +3 standard deviations $\left(\bar{X}\pm 3SD\right)$ of the OD values of the negative control plasma samples [17]. Thus, a sample was considered positive if the OD value exceeded the cutoff value. Sensitivity, defined as the percentage of individuals with a given condition whom the assay identifies as positive for that condition [28], was calculated as follows,

$$Sensitivity=\frac{N_{tp}}{\left(N_{tp}+N_{fn}\right)}$$

where *N_tp_* and *N_fn_* denote the number of true positive results and the number of false-negative results, respectively. In this paper, we define true positives as plasma samples collected from rats confirmed as having *A. cantonensis* infection through necropsy. False negatives are defined as plasma samples confirmed as having *A. cantonensis* infection through necropsy but whose OD value fell below the positive cutoff. True negatives are defined as plasma from uninfected rats. Spearman’s tests were used to measure correlations of absorbance (OD levels) with rat sex and weight, the staining of larvae fed to rats, and the number of adult worms found during necropsy, for each antibody isotype. All analyses were performed using Minitab Statistical Software v21.3.

## 3. Results

**Figure 1:**
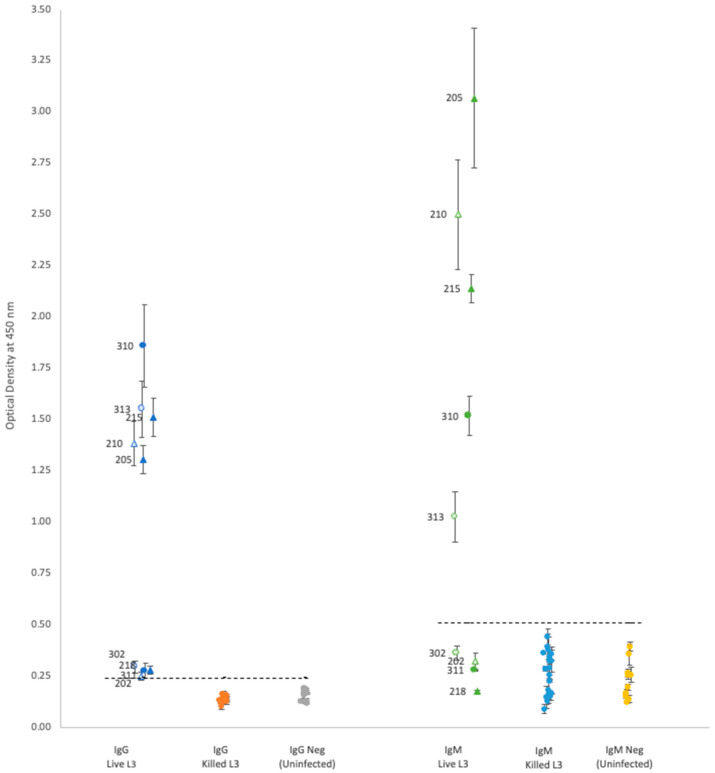
A comparison of optical density (*Y*-axis) by experimental groups (*X*-axis) using IgG and IgM conjugate, respectively. IgG and IgM are comparable due to their common assay concentrations; primary antibody concentration (1:1000), secondary antibody concentration (1:5000), and antigen concentration (0.25 µg/well). Individual rat ID numbers are as indicated. Dashed lines indicate positive cutoff. ▲ = Male rats fed stained live L3, △ = Male rats fed unstained live L3, ● = Female rats fed stained live L3, ○ = Female rats fed unstained live L3.

**Figure 2:**
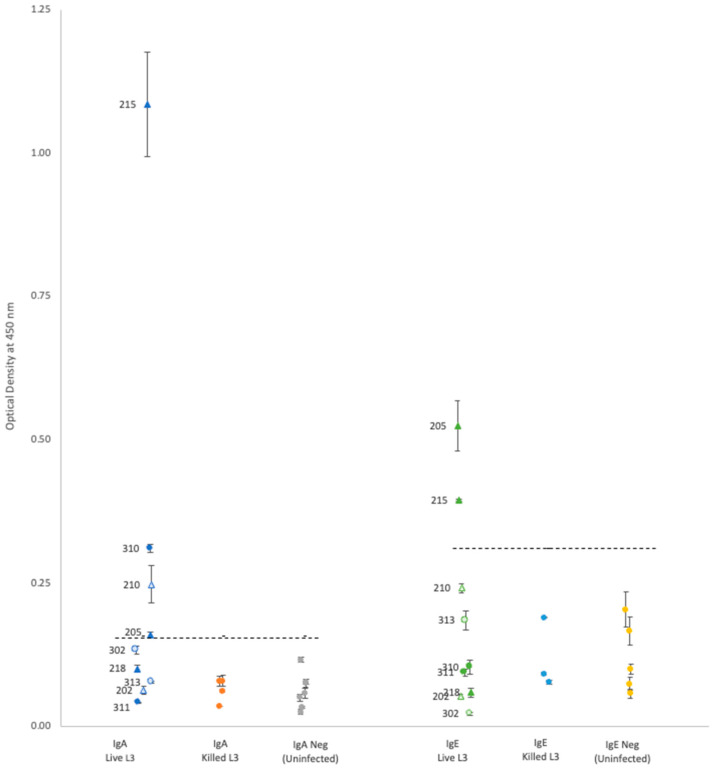
A comparison of optical density (*Y*-axis) by experimental groups (*X*-axis) using IgA and IgE conjugate, respectively. IgA and IgE are comparable due to their common assay concentrations; primary antibody concentration (1:50), secondary antibody concentration (1:1250), and antigen concentration (0.5 µg/well). Individual rat ID numbers are as indicated. Dashed lines indicate positive cutoff. ▲ = Male rats fed stained live L3, △ = Male rats fed unstained live L3, ● = Female rats fed stained live L3, ○ = Female rats fed unstained live L3.

For each isotype, optical density (OD) results showed that neither propidium iodide staining nor rat sex status produced significant differences within the groups of rats gavaged with either live or killed larvae (Table 1 and Figure 1 and Figure 2); thus, the results are discussed as simply live vs. killed.

Plasma from rats gavaged with live larvae (9 rats) generally showed markedly higher OD values than those from rats gavaged with killed larvae (20 rats) or the uninfected control group (10 rats). However, using the mean of the control group +3 standard deviations as a cutoff value for determining positive vs. negative infection status provided different results for each antibody. Positive OD cutoff values for IgG, IgM, IgA, and IgE were 0.238, 0.505, 0.156, and 0.309, respectively (dashed lines in Figure 1 and Figure 2). This positive cutoff value resulted in a sensitivity for each isotype of 100%, 56%, 44%, and 22%, respectively. The mean OD values for rats fed killed larvae all fell below the positive cutoff in each isotype, indicating their negative infection status.

Each isotype showed a wide range of OD readings in the plasma of rats fed live larvae; however, no significant correlation (Spearman’s test) was detected between ELISA OD levels and the rat’s proportional weight difference, or the number of adult worms found in the rat’s lungs upon autopsy regardless of antibody isotype (Table 1).

## 4. Discussion

The results of this study confirm the presence of IgG, IgM, IgE, and IgA isotypes against the Hawai’i 31 kDa isolate in at least some six-week PI rat plasma, with wide variation in the sensitivity of each isotype. The 22–100% range in sensitivity observed in this study differs from human studies conducted with crude antigen, which found each isotype had a roughly 70–98% sensitivity during acute infection [16]. Additional research is needed to determine the cause of this discrepancy. Similarities in the humoral immune response exist across different antigens, hosts, parasite burdens, diagnostic methods, and even infections with different *Angiostrongylus* species. For example, studies using a variety of diagnostic methods, parasite burdens, and stages of *A. cantonensis* for crude antigen preparation have shown that during primary infection, total antibodies and the IgG isotype against crude *A. cantonensis* antigens can be detected in rat sera as early as 10–30 days PI and peak at 50–60 days PI [19,20,21,22]. Our study found 100% sensitivity of IgG against the Hawai’i 31 kDa isolate at 42 days PI, which correlates with this detection window. Similarly, serum antibodies in canine *A. vasorum* infections are also detectable at 27 days PI and peak at 55–57 days PI and remain detectable at 83–84 days PI [29]. The consistency of antibody detection across different *A. cantonensis* antigens, different *Angiostrongylus* species, and in different hosts, indicates that IgG against the Hawai’i 31 kDa isolate may be a good biomarker for NAS infections of accidental hosts as early as 10 days PI through at least 60 days PI, possibly longer. Additional research is needed to determine the sensitivity of the IgG-Hawai’i 31 kDa ELISA assay throughout this potential testing window.

Given that early treatment may result in better patient outcomes, possibly avoiding chronic sequelae, a laboratory diagnostic that can detect NAS acute infections within the symptom onset window could result in better patient outcomes [9]. Presentation of NAS symptoms may occur before the potential IgG testing window described above, with humans exhibiting symptoms between 7 and 21 days PI [9] and canines showing symptoms at approximately 11 days PI [30]. While NAS infections have been confirmed in equines, the time of symptom onset is unknown [30]. Although Takai et al. [20] found both IgG and IgM antibodies in rats at 10 days PI, this early detection has not been confirmed, with Kanbara et al. failing to detect an IgG antibody response in rats until 20–30 days PI [21]. Given the IgM isotype is the first to appear in a primary infection, and our results showed relatively high OD readings in 56% of rats gavaged with live larvae, the IgM isotype against the Hawai’i 31 kDa isolate could be an informative laboratory diagnostic during NAS symptom onset period. Unfortunately, it seems the change in IgA or IgE titers over time in NAS infections has not been investigated. While our study was able to detect IgA and IgE in some rats gavaged with live larvae (44% and 22%, respectively), additional research is needed to determine if the IgA or IgE isotypes against the Hawai’i 31 kDa isolate could be informative for NAS infections.

Our results showed distinctive bimodal clustering in IgG and IgM OD values (Figure 1) for rats with active infections, but the reasons for this are unclear. Although two studies have found that the total antibody concentration, IgG and IgM, in rat serum varies according to larval load at time of infection [20,21], the positive plasma used in this study came from rats that were all gavaged with 50 live larvae. Our results differ from ELISA results of active human NAS infections, which show a relatively uniform distribution pattern in OD values [16]. Moreover, no correlation was found between titers and the number of adult *A. cantonensis* found in rat pulmonary arteries. Additional research is needed to determine what causes the variation in isotype OD values in the Hawai’i 31 kDa ELISA assay.

In our study, there was no detectable antibody response in rats fed killed larvae. However, this might be a consequence of the methanol used to kill the larvae, which may have denatured the 31 kDa proteins on the surface of the larvae. Thus, additional research is needed to determine under what conditions dead larvae can generate a humoral immune response.

This study is the first step toward a more in-depth understanding of the humoral immune response against the *A. cantonensis* 31 kDa antigen. As seen with *A. vasorum* [29], it might be possible that blood antibody titers using ELISA offers a wider window than other high-sensitivity laboratory diagnostic methods such as real-time PCR of blood, and thus warrant further investigation.

## Figures and Tables

**Table 1:** Pairwise Spearman Correlation (*r*).

	Mean OD: IgG	Mean OD: IgM	Mean OD: IgA	Mean OD: IgE
Stain	−0.066	0.045	0.206	0.193
Sex (all rats)	0.048	−0.107	−0.407	−0.265
Proportional weight diff.	−0.317	−0.017	0.033	0
Total worms in rat lungs	−0.038	−0.5	−0.517	−0.167

## Data Availability

All data supporting the reported results are provided in the manuscript.

## Supplementary Materials

The following supporting information can be downloaded at: https://www.mdpi.com/article/10.3390/pathogens12040625/s1, Table S1: Supplementary Data-DAAutopsy vs. OD.xlsx.
